# Supplementary material for: Characterization of Stem-like Circulating Tumor Cells in Pancreatic Cancer
Source: Diagnostics (Basel). 2020 May 15;10(5):305. doi: 10.3390/diagnostics10050305 (PMC7278018; doi:10.3390/diagnostics10050305)
Supplement: Supplementary file 1 [file diagnostics-10-00305-s001.pdf]

SUPPLEMENTARY MATERIALS

Figure S1. Pre-processing the raw data of the pancreatic circulating tumor cells (CTCs). (**A**) Total read counts of single-cell RNA sequencing data of 75 murine pancreatic CTCs. The red horizontal line represents the cutoff, two counts per million (CPM). Three samples below this number were excluded. (**B**) Distribution of transformed data using a boxplot. The figure is generated by iDEP 9.0 (http://bioinformatics.sdstate.edu/idep/).

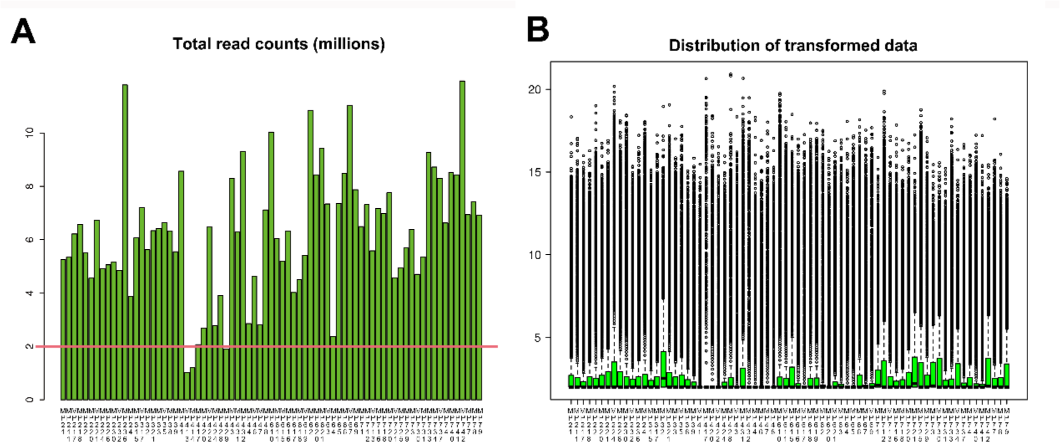

Figure S2: The classification of 72 samples. (**A**) Principal component analysis (PCA) scores plot of 72 samples. In this figure, x and y axes represent the principal component 1 (PC1) = 16.6% variance and PC2 = 6.1% variance, respectively. All samples could be divided into three distinct clusters. The corresponding ellipses were plotted based on a 95% probability from the same group. The figure is generated by ClustVis (https://biit.cs.ut.ee/clustvis/). (**B**) The heatmap of 3000 genes expression. The relative color scheme was established, in which the maximum values were converted to red and the minimum convert to green in each row. We used the Pearson correlation as a metric to do the hierarchical clustering with average linkage in both columns and rows. The visualization was done by the MORPHEUS (https://software.broadinstitute.org/morpheus/).

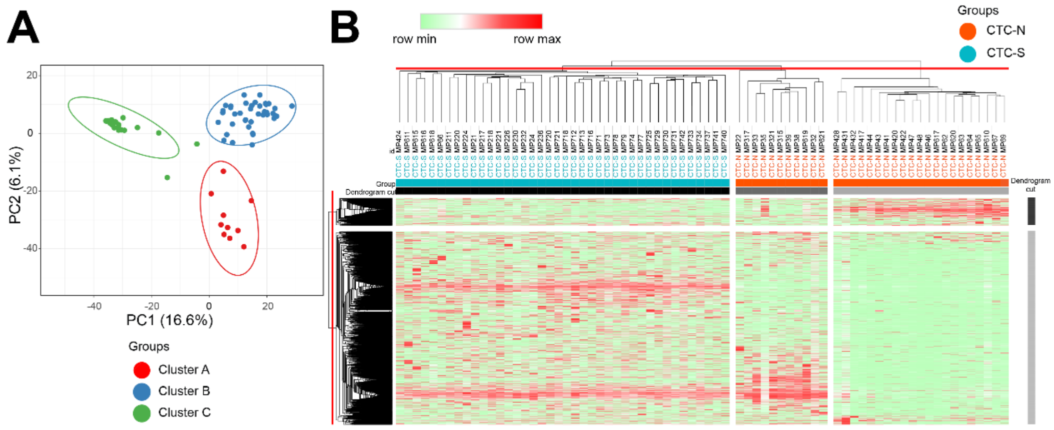

Figure S3: The differentially expressed genes. (**A**) The volcano plot of differentially expressed genes. Genes with ∣Fold change (FC)∣ > 2 and a corrected p-value, false discovery rate (FDR) < 0.050 (Benjamini-

Hochberg procedure) were defined as differentially expressed genes. Red dots represent up-regulated genes and blue dots down-regulated genes. This plot was generated by iDEP (http://bioinformatics.sdstate.edu/idep/). (**B**) The Venn plot shows the intersection between the top 3000 most varaible genes and differentially expressed genes. We utilized imageGP website (http://www.ehbio.com/ImageGP/index.php/Home/Index/index.html) to plot this.

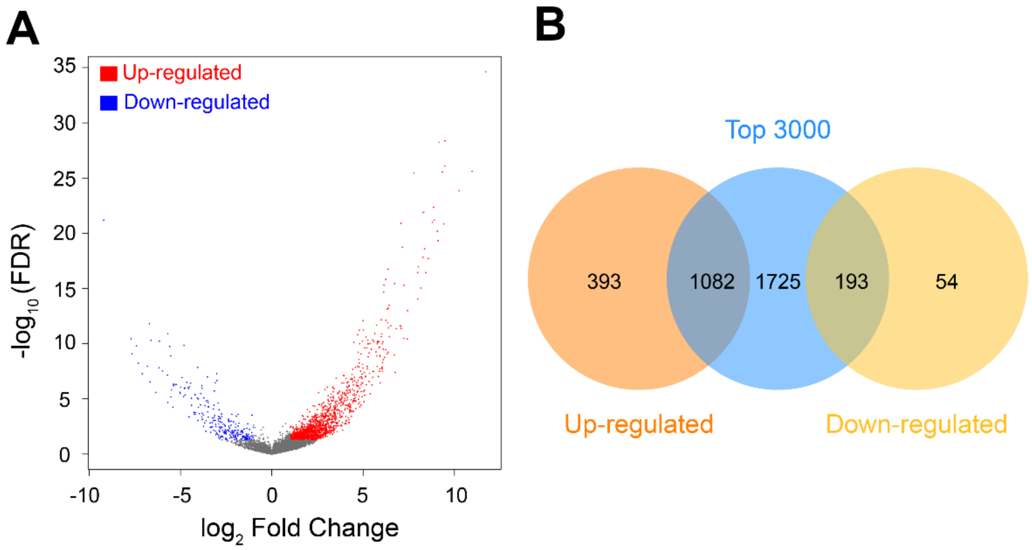

Figure S4: The construction of the weighted gene co-expression network of 72 pancreatic CTCs. (**A**) We clustered all 72 samples with the Euclidean distance. (**B**) Analysis of the scale-free fit index and the mean connectivity for various soft-thresholding powers. The power of 4 (R^2 = 0.93) is the lowest soft thresholding for the scale-free topology network.

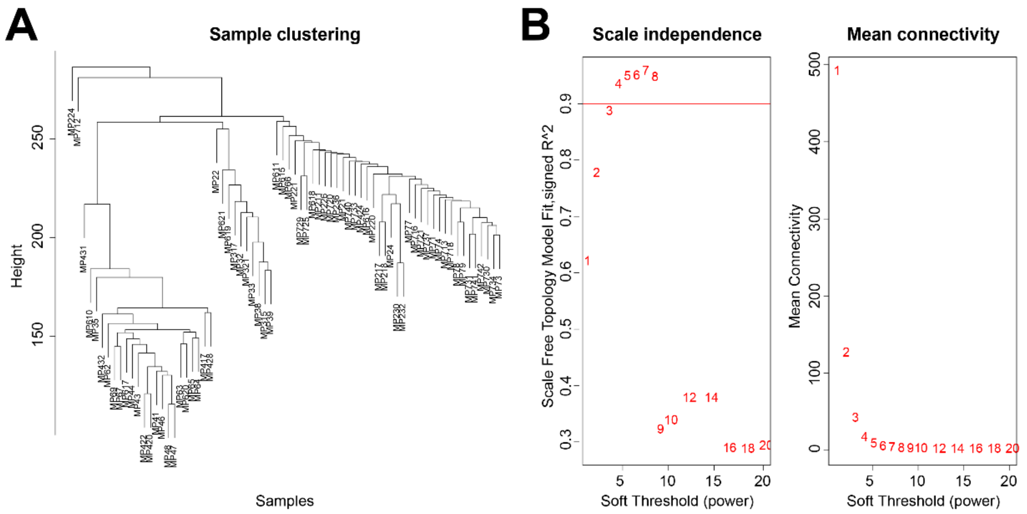

Figure S5: The heatmap of genes involved in the adherens junction pathway. MORPHEUS- generated heatmap showing the expression of the marker set in CTC-S and CTC-N subgroups. The relative color scheme was established, in which the maximum values were converted to red and the minimum convert to green in each row. The marker names, groups, log_2(Fold Change) values, and false discovery rate (FDR) values were listed on the right side.

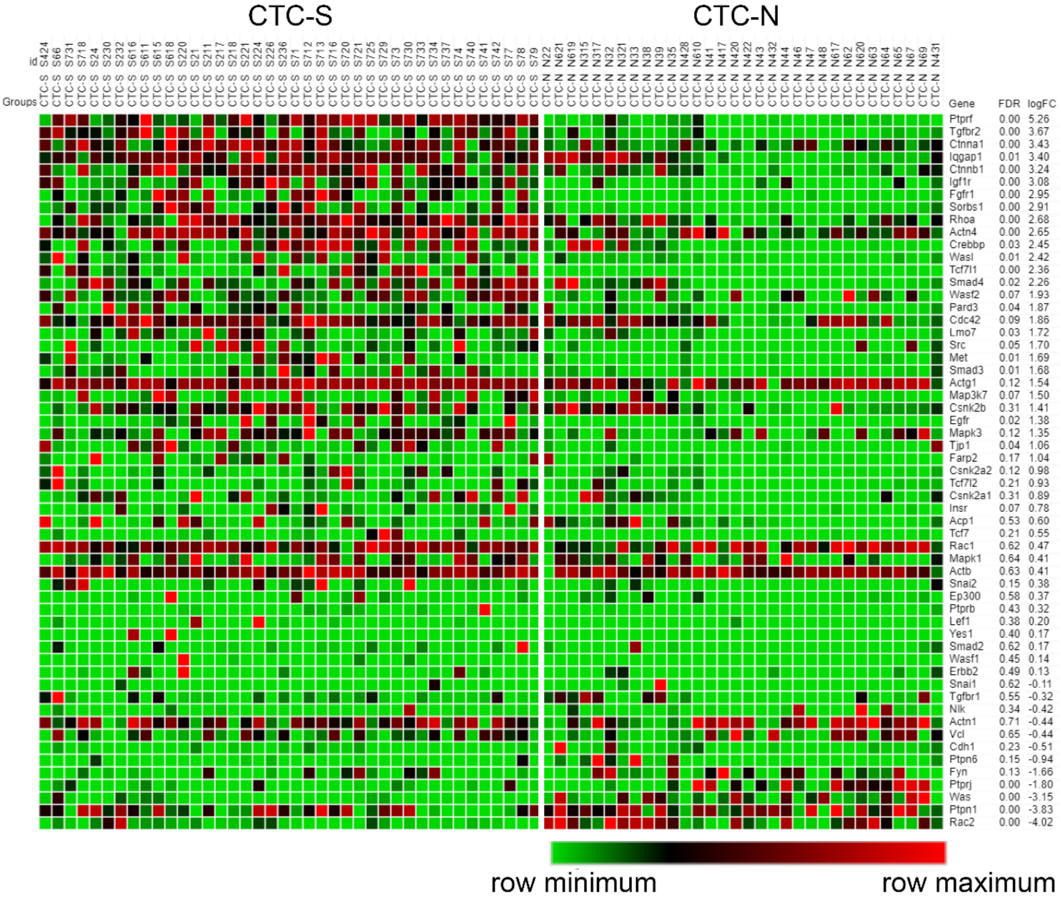

Figure S6. Correlation analysis of *Ctnnb1* and *Wnt* family members in the pancreatic CTC-S group. Histograms of gene expression are on the diagonal, and the lower-left part was the scatter plots and the numbers in the upper right part was the Pearson's correlation coefficient. ***, FDR < 0.001; **, 0.001 < FDR < 0.010; *, 0.010 < FDR < 0.050; ., 0.050 < FDR < 0.100.

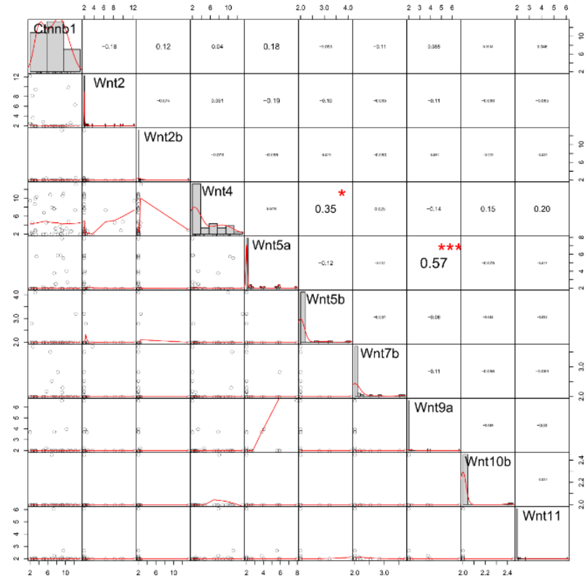

Table S1. Online web tools utilized in this study

Online Tool Name	URL	Date (mm/dd/yy)
ClustVis	https://biit.cs.ut.ee/clustvis/	03/07/2020
g: Profiler	https://biit.cs.ut.ee/gprofiler/gost	03/23/2020
GeneCards	http://www.genecards.org/	03/31/2019
GEO	https://www.ncbi.nlm.nih.gov/geo/	08/19/2019
GEPIA 2	http://gepia2.cancer-pku.cn/#index	03/07/2020
iDEP.90	http://bioinformatics.sdstate.edu/idep/	03/07/2020
imageGP	http://www.ehbio.com/ImageGP/index.php/	04/23/2020
KEGG	https://www.genome.jp/kegg/	08/19/2019
MORPHEUS	https://software.broadinstitute.org/morpheus/	03/07/2020
PATHVIEW	https://pathview.uncc.edu/home	08/19/2019
ProcessOn	https://www.processon.com/diagrams	03/23/2020
STRING 11.0	http://string-db.org/	08/19/2019
TCGA	https://cancergenome.nih.gov/	08/19/2019
UniProt	https://www.uniprot.org/	08/19/2019
